# Editorial: Statistical methods, computing, and resources for genome-wide association studies, Volume II

**DOI:** 10.3389/fgene.2022.1040022

**Published:** 2022-10-20

**Authors:** Lide Han, Hailan Liu, Guolian Kang, Min Zhang, Riyan Cheng

**Affiliations:** ^1^ Vanderbilt Genetics Institute, Vanderbilt University Medical Center, Nashville, TN, United States; ^2^ Maize Research Institute, Sichuan Agricultural University, Chengdu, China; ^3^ Department of Biostatistics, St. Jude Children’s Research Hospital, Memphis, TN, United States; ^4^ Department of Statistics, Purdue University, West Lafayette, IN, United States; ^5^ Department of Psychiatry, University of California San Diego, San Diego, CA, United States

**Keywords:** genome-wide association studies (GWAS), gene-based testing, longitudinal phenotypes, binary phenotypes, sparse mixture-of-experts models

As in the previous volume, this collection presents several research articles that address statistical methodology, computing, and resources for genome-wide association studies (GWAS). The research interest covers association studies of longitudinal data, multiple testing, computationally efficient analysis of binary phenotypes and more.

Phenotypic data is often longitudinal in plant science. Genetic analysis of longitudinal phenotypic data can treat data at different time points separately or fit a growth curve that depicts the developmental process. The latter is commonly practiced at single genetic variants while gene-based GWAS are often of interest. Li et al. propose a function-on-function regression method, a longitudinal functional data association test, which not only models the developmental process but also takes advantage of gene-based testing, and shows that their method performs well for the identification of genetic variants switching in the growth and development stage.

Proper controlling of the type I error rate is an important issue in GWAS. While the permutation test is regarded as the gold standard, its extensive computation is prohibitive in genetic studies which often include thousands of samples and millions of single nucleotide polymorphisms (SNPs). In human genetics, a broadly-accepted *p*-value cutoff is commonly used for statistical inference. However, the genome size and the SNP distribution often vary from study to study, and it is thus desirable to determine data-driven significance thresholds. Many computationally efficient methods have been proposed for the derivation of genome-wide thresholds, including Bonferroni correction based on an estimated effective number of tests and Brown’s method, which take the SNP dependency into account to achieve a higher power while properly controlling the type I error rate. Cinar and Viechtbauer survey several gene-based testing methods and evaluate their performance *via* simulation studies. Their work provides valuable information to those who seek computationally efficient methods for significance threshold estimation in GWAS.

Categorical phenotypes are not rare in genetic studies. Genome-wide association studies of categorical phenotypes are computationally challenging due to the incorporation of genetic relatedness matrices (GRM). The most common categorical phenotypes are binary. There have been enormous efforts to address the computational issues in GWAS with binary phenotypes. Strategies include the use of spare GRM plus Saddle Point Approximation. With a focus on computational efficiency and the control of false positives, Gurinovich et al. empirically evaluate several known software programs such as SAIGE and fastGWA-GLMM and provide food for thought for researchers who consider software programs that perform GWAS analysis with binary phenotypes.

It is known that many diseases are heritable. Genetic data not only plays an important role in the identification of genetic factors underlying diseases but also is helpful for determining disease subtypes. Courbariaux et al. study phenotypic data that is longitudinal and arises from multiple sources, and propose a sparse mixture-of-experts model that incorporates genetic information for disease subtyping. The authors claim two advantages of their methods over others. First, their model-based clustering utilizes original data without transformation and thus facilitates the interpretation of results. Second, they address the large-scale problem due to massive genetic data by model selection. This collection also touches on two popular problems, heritability estimation and polygenic risk scores (PRS). Heritability is often reported in GWAS. Zhang and Sun show that heritability tends to be overestimated in the presence of Hardy–Weinberg disequilibrium. Such information should be useful for those who are interested in heritability estimation. As genomic prediction, PRS concern estimation of genetic values underlying a trait of interest. Zhang et al. compare methods that utilize sex-specific PRS and share useful information with interested readers.

To sum up, this volume highlights several research interests in statistical methods, computing, and resources for GWAS which we hope contribute to the society of genetic studies.

